# The function of episodic memory in animals

**DOI:** 10.1098/rstb.2023.0403

**Published:** 2024-09-16

**Authors:** Susan D. Healy, T. Andrew Hurly, Jeanne Godard, Maria Tello-Ramos

**Affiliations:** ^1^ Centre for Biological Diversity, School of Biology, University of St Andrews, St Andrews KY16 9TH, UK; ^2^ Department of Biological Sciences, AWESB, University of Lethbridge, 4401 University Drive, Lethbridge, Alberta T1K 3M4, Canada; ^3^ Department of Biological Sciences, Macquarie University, Sydney, New South Wales, Australia

**Keywords:** wild, natural selection, episodic memory, what–where–when, what–where–which

## Abstract

The best-known example of episodic memory in animals came from food-storing birds. One of the beauties of the food-storing system was that inherent in the behaviour were the elements that (at the time) made up episodic memory: what, where and when. While there were then already plenty of data on animals’ ability to put together what and where, the addition of the time element in animals’ memory and its testing was one that was both new and experimentally challenging. It has, however, led to an increasing variety of examples showing that animals can put together all three informational components. If episodic memories can be described as those memories that make any one of us who we are, why should non-human animals have such memories? Here, we argue that episodic memories play a significant functional role in the lives of real animals, in particular, enabling them to make decisions about how they might or should act in their future. We support our argument with data from a range of examples, focussing on data from the field.

This article is part of the theme issue ‘Elements of episodic memory: lessons from 40 years of research’.

## 1. Natural selection and the value of episodic memories

We tend to think that episodic memory is the pinnacle of the types of memory, that it is the most complex, and that although typical to humans, there may be only rare examples of animals with such memories. However, perhaps we might consider that it is the most simple, the basic building block for all of the kinds of memory that are useful to us and to other animals. The memories built on top of an episodic memory are useful because they are associative, semantic or other, but they generally (perhaps always) require more than one experience to come into being. They enable us to generalize from the specific: from a single experience into multiple experiences from which we and other animals extract the commonalities across those episodes. Here, we pursue this train of thought by considering the plausibility of this notion. We also consider whether there might be selective value to having episodic memories. We do this by examining evidence for episodic memories in animals in the wild, because it is here, rather than in the laboratory, that natural selection acts.

We begin our review of episodic memories in wild animals with a consideration of the putative selective value of being able to remember specific episodes. It will be conspicuous that we do not raise the issue of autonoetic consciousness or the personal experience of a memory [1], the component of episodic memory that renders it impossible to demonstrate in non-human animals, no matter how hard researchers might try [2]. We are far from the first to recognize the problem of autonoetic consciousness and the search for episodic memory in animals (e.g. [3,4]). However, we consider the endeavour has led to uncovering a variety of memory capabilities that may have remained hidden without the incentive of attempting to find the pot at the end of the episodic rainbow. Those capabilities include the integration of what–where–when and of what–where–which, both of which have been found in a diverse range of animals. We do not intend to survey that literature but rather to first look at the possible selective value to retaining something like an episodic memory, then to look at supporting data from animals investigated/tested in the wild, and finally, to encourage further similar efforts. One of our intentions is to provide food for thought, even to extend that to provoking reconsideration of some of the prevailing views of episodic memory.

Much effort has gone into thinking about the value of episodic memory for enabling future planning (e.g. [5–7]). This has tended to take the form of looking to see what animals do now in order to behave appropriately in the future, such as collecting objects that can be used as tools when those tools might enable food extraction or for throwing at others (e.g. [6,8]). We can see this when food storers move food items from one cache site to another if they have been observed during their caching [9]. However, perhaps we might first ask what value there is to keeping any kind of memory if it is not to aid in future decision-making. Even if we humans might learn information for the fun of learning, it seems a little less likely that learning for fun has been a major selective pressure for animals in the wild. Following this logic, what might be the selective value for humans to have episodic memories? We think about episodic memories as being central to the definition of ourselves such that when we begin to lose them owing to dementia, we lose something of ourselves. Is this likely to be the situation for other animals, and if so, how would this be favoured by natural selection? Although we humans chat a lot with each other about our episodic memories (and thereby reconstruct them over and over), it does not mean social glue or similar is or was their key evolutionary value. More importantly, it does not mean that there is very much of a selective value at all. Is our fitness enhanced because we can remember specific episodes, i.e. do episodic memories enable humans to have more offspring? Might fitness be enhanced by having the capacity to put together the components that make up an episodic memory? What if we consider the product (the episodic memory) and the process (information integration) separately: is the process the useful focus for natural selection, the product or both? Perhaps these memories produce the raw materials that allow us to model possible future behaviours.

It seems plausible that every memory is initially that of an episode, but parts of that memory get dropped or are not reinforced as they become less valuable/salient. If different parts to that memory were laid down initially with different salience to each component, this ‘dropping off’ would be a regular event. The result would be, for example, that while one knows (remembers) that Paris is the capital of France, one has forgotten the geography classroom in which this fact was learned, or that particular geography class that happened on a Monday in the last week of term when you were nine, and you were excited about the impending holidays. With no obvious benefit to remembering all of the details of this experience, indeed, perhaps you had to be told more than once that Paris was the capital of France because it was not an especially interesting piece of information for you. A once-episodic memory becomes a semantic memory. Similarly, did your cat develop her habit (a different kind of memory) of asking for play in your office at 14.00 because her first experience of play (what) at 14.00 (when) in your office (where) was, for her, especially rewarding? This led her to ask for play in your office at 14.00 the following day, and because the first instance occurred during COVID−19, she was again rewarded and so on. In this case, what has become a habit, began from a single event that occurred in a specific place at a specific time. In this case a positive experience, with the location and time of day plus the activity subsequently regularly reinforced. Certainly, your cat has put together (integrated) multiple informational components into an event she attempts to recreate, and you will have multiple examples how she can do this. For example, for your cat to receive treats, she has to ask for them in the kitchen at 19.00; again, integration of a what, a where and a when leads this cat to be rewarded. This different integrated memory is another that does not remain as an episodic memory but becomes an example of associative conditioning or even semantic memory as the integrated information becomes a singular fact. Also, she may even remember the very first time she received play in the office or treats in the kitchen, if you could only ask her. This is a question the asking of which remains a job for Dr Dolittle, however.

One might also imagine examples where the experience was not positive, such as an interaction with a predator in which the prey animal survives. It seems very probable that such an encounter would be highly salient and memorable and lead to the survivor avoiding not just the place, but also possibly at the time of day at which the encounter occurred. Remembering both the when and the where this experience happened would be hugely beneficial, and even more so would the inclusion of memory for the identity of the predator. After all, some predators are more dangerous, some have stronger site fidelity, and so on. Unlike the cat learning to beg for play (rather than food) at a specific time and place, the surviving prey animal does not want, or need, a second experience for the event to be memorable, and for that event to shape its future behaviour. This future may or may not involve specific planning, or certainly not planning in the way that is currently accessible to researchers. It is highly likely that this future would involve avoidance of the location of the encounter, perhaps with some kind of time stamp. Depending on the salience of the encounter, the prey animal might remember and continue to avoid this location for the rest of its life. It may pass on this information to others, including its offspring. From a single episode, information may also be passed through generations as memories of other kinds, e.g. bird songs are transmitted (cultural evolution). Alternatively, a single episode may form the basis for a lasting avoidance of particular kinds of locations at particular times of day. Importantly, this example is one in which an episodic memory has significant fitness value: the surviving prey lives to experience another day and is more prepared not just to survive future interactions but will waste less time fleeing from a predator’s regular haunt.

Which memories of this kind are going to be selectively valuable will be related to the salience of the memorable event: episodes of especial importance should be those that are much more readily remembered. As to the number of episodic memories that should be kept, even if we humans gain socially from remembering lots of episodic memories, it is less obvious that this is true for animals, with perhaps exceptions of species like food storers. Food storers may store many hundreds of items and do so relentlessly throughout their lives. However, unlike human episodic memories, these memories for food stores should be one-time-use memories and once the food is retrieved, the memory for the location from which the food store was retrieved ideally should be at the very least, updated, or even better, deleted. This one-time use feature of these memories means they differ quite considerably to the potentially often-revisited episodic memories we humans might treasure. They also differ from the putative episodic memories that would derive from a prey’s successful avoidance of a predator. This latter memory would be much more similar to a human’s episodic memory, as it could be reconstructed and revived, revised and refined and even shared with others. They may or may not number as many as the human episodic store, but by the keeping in perpetuity, they be very similar in kind.

## 2. Problems for testing for the existence of episodic memory

If one allows that there are plausible instances in which animals might benefit from retaining episodic memories, one might ask why we felt we needed to provide a narrative for the selective advantage of possible episodic memories. This is because there are, in fact, rather few agreed-upon instances of episodic memory in animals. Other authors have very clearly described thoroughly and elegantly how most of the extant examples could be explained by some other mechanism than episodic memory (in particular [10] and other related papers). This being the case, a paper on such memories in wild animals would be extremely short. Indeed, non-existent. There are two major causes for the lack of a list of examples. Firstly, although there are prominent examples of episodic memory from primates and birds, especially from the work of Nicky Clayton, Tony Dickinson and colleagues, the search for episodic memories has tended to focus on rodents tested in the laboratory. This selectivity itself has largely come about for two reasons; the most conspicuous is the strong emphasis on the value of rodents as a model for humans, both in multiple neuroscience contexts and the associated medical value: the prize outcome for most of these researchers would be to find a way to undo the damage caused by Alzheimer’s disease. Alzheimer’s specifically affects our ability to recall episodic memories, and the impact of this loss is considerable on the way we live our lives. For these researchers using rodent episodic memory as a model in this way, a comparative library of episodic memory examples holds no especial appeal.

Secondly, and the reason that laboratory rodents are one of the main models for human neuroscience, is the logistical advantage of using them. Beyond the value of working with an animal system about which so much is known, for which there are, or soon will be, pertinent genetic mutants and other useful resources, work by Crystal [11,12], Panoz-Brown *et al*. [13] and Zhou & Crystal [14], in particular, has provided an enormously valuable platform for directing examination of episodic memory, and not just in rodents. The very thorough examination of the performance of rats on tasks that may actually test some other, very similar memory ability is exemplary, although for some, it will be intimidating (e.g. [10]).

The other reason for the lack of a list of examples of episodic memory in wild animals is because of the problem in identifying a suitable experimental system that is not rodent-based and that is testable in the wild. Clayton & Dickinson’s [15] key observation that the food-storing behaviour of some bird species could provide an experimental setting for examining episodic memory was inspired. However, that hugely influential work did not lead to a flowering of experiments on other food-storing species (of which there are not lots anyway), not least because the requisite manipulations look as if they need to be conducted in the laboratory. At least, that is, if one wants to be very sure of the kind of memory that one is labelling episodic memory. Crystal and colleagues’ work has shown the care that must be taken to exclude a variety of possible alternative interpretations to that of episodic memory (e.g. controlling for exposure time and/or familiarity of a stimulus [10]. To conduct such tests in the laboratory requires the capture and holding of a population of food storers, most probably birds, but could be rodents. There was a small flowering of laboratories in the UK and North America working on spatial memory in avian food storers in the 1990s, but with retirements of key researchers and the increases in cost, the appropriate holding and testing facilities have diminished and are now increasingly few and far between. An advance on the work done in the 1990s is that data are coming in from the field on memory in food-storing birds, both mountain chickadees *Poecile gambeli* and toutouwai *Petroica longipes* (e.g. [16–18]). However, these efforts are directed largely at quantifying specifically spatial memory and at the selective advantage to possessing better spatial memory (e.g. [19–22]), rather than examining episodic memory. One reason might be that for all the difficulty of investigating episodic memory in the laboratory, even in rats, it is very much more difficult to conduct the appropriate tests in the field [23].

Looking for, or investigating, episodic memory in laboratory animals is challenging enough, but to do so in the wild is, in today’s parlance, ‘next level’. However, given that it has been found in laboratory rats and scrub jays tested in the laboratory, just as rodents can serve as a model for human memory, they can themselves serve as a model for memory in other vertebrates. If rodents share sufficient neural similarity with humans, then they also do so with other vertebrates: they share many of the basic neural features required for experiencing episodes, laying down memories of this kind and retrieving them. That said, evidence of episodic memory from cephalopods, including cuttlefish and, more recently octopus [24–26] shows that animals which do not have a brain remotely like that of humans, or other vertebrates, are still capable of episodic memory. This breadth of taxonomic diversity might suggest that this kind of memory has very broad selective value in the sense that it has evolved independently in multiple taxa. Alternatively, this kind of memory may have arisen early in animal evolution and consequently is taxonomically broad because it has an important narrow function in many subsequent taxa.

## 3. Steps towards examining episodic memory in the wild

Even if the current status of the debate over episodic memory means that it is not possible to conduct the necessary experiments in the wild, there are data from the field that are strongly suggestive that episodic memory occurs in many more species than humans, rodents, scrub jays and cephalopods. We consider that the experiments conducted by Marshall *et al*. [27] and Jelbert *et al.* [28] are among those that have come closest to showing episodic memory in the field. Both sets of experiments were predicated on the foraging biology of territorial hummingbirds. Territorial hummingbirds as a group of animals to examine makes sense in a way not dissimilar to the value of using food-storing birds and relying on a behaviour naturally performed. This is because a territorial hummingbird foraging on flowers would usefully remember the locations of the flowers that he has recently emptied so that he can avoid them, at least for a duration that would be long enough for the flower(s) to refill. This means remembering not just where the flowers are, but also when they had been visited (e.g. [29,30]).

**Figure 1.**
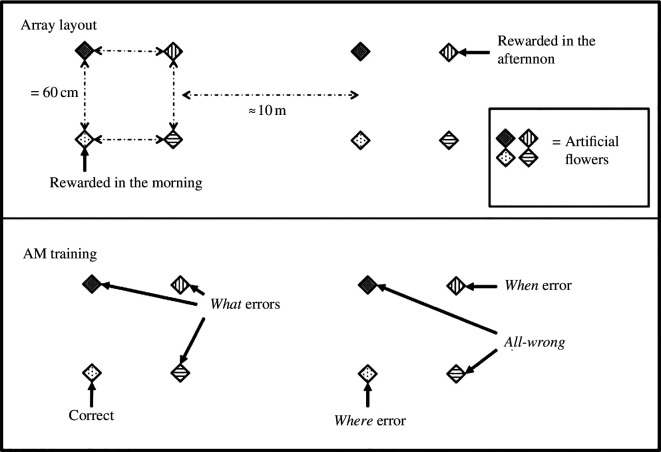
In the upper panel is a schematic showing an array of eight artificial flowers, arranged in two squares. The colour patterns of the flowers and their relative position in each of the sets of four flowers were the same. The lower panel, labelled AM training, shows the categorization of the type of visit a hummingbird might make to each of the flowers. Only one flower contained the reward (the correct flower), and visits to the other flowers were categorized as *what, where, when* or *all wrong* errors. From Marshall *et al*. [27].

Both experiments involved wild, free-living hummingbirds that had been trained to forage from artificial flowers, which contained small amounts of nectar. In the Marshall *et al*. experiment, the birds were presented twice a day (in the morning and the afternoon) with two arrays each of four coloured flowers. In the morning presentation (somewhere between 8.00 and 10.00), the rewarded flower was, for example, the blue flower in the left-hand array, while in the afternoon (somewhere between 14.00 and 16.00), the reward was to be found in the yellow flower in the right-hand array (figure 1). For each time of day, just one flower contained reward. Birds were allowed to visit the arrays up to six times a session and for up to seven days. The data that were analysed came from just the first visit on each day. Already, it will be clear that these birds were not being tested for their episodic memory capacities. Rather, the experimental set-up was designed to be able to examine the three components, that for non-human animals, are considered to constitute episodic memory: the what (colour), the where (flower location) and the when (time of day). Although the hummingbirds were most likely to return to the flower identified by all three components together, the pattern of mistakes the birds made showed that the location was the best remembered of the three components, which fits well with what we know of hummingbird memory from many experiments (e.g. [31–34]). Choosing the flower of the wrong colour (but at the correct time in the correct location) was the most easily corrected mistake as it was nearly always followed by visiting the correct flower. The most common mistakes were those pertaining to the ‘when’ component and these were not readily put right. The difficulty with getting the ‘when’ right remains a challenging component of demonstrating episodic memory in non-human animals (e.g. [35,36]). It turns out that memory for when is not without its challenges for humans either (e.g. [37,38]).

For this reason, what–where–which context has been used as an alternative method of asking if animals can combine the three key elements of this kind of memory. This method has been applied to testing rats, exploiting the tendency of rats to explore novel objects or familiar objects in novel locations in preference to familiar objects in familiar locations (e.g. [39–41]). The objective was to focus on the ability of an animal to remember an occasion, which need not be about time *per se*: key is that the specific episode is identifiable among other similar events. A related version of this task, the novel object recognition task, has been applied on occasion to animals in the wild but typically more in the context of neophobia (e.g. [42]) or personality assays (e.g. [43]), rather than with especial interest in memory capacities. Importantly, if the temporal component of an episodic memory is challenging to discern, then it might be easier to look for a non-temporal component that serves a similar function.

**Figure 2.**
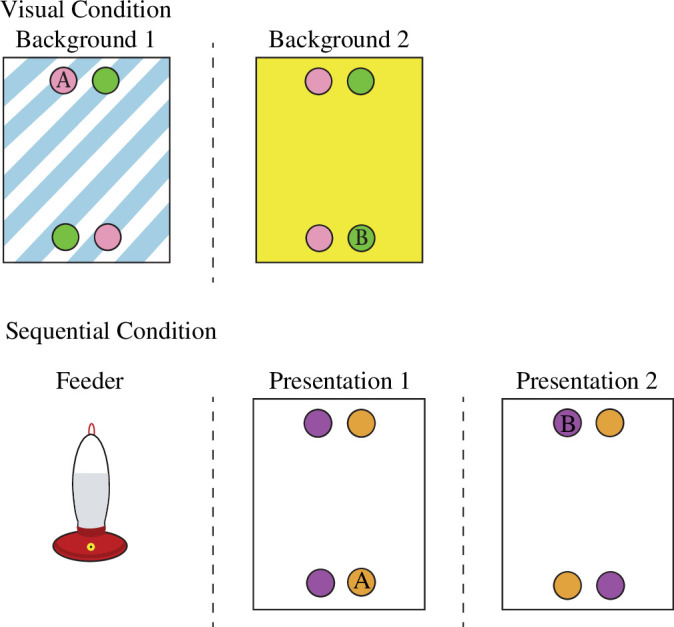
In the upper section is a schematic from the visual condition of the Jelbert *et al*. [28] experiment. Each board was coloured with a different colour pattern and only one of the four flowers on each contained reward. On the board labelled background 1, the correct (i.e. the one containing reward) flower was the pink one on the top of the board, whereas on the board labelled background 2, the correct flower was the green one at the bottom of the board. In the lower section is a schematic showing the sequence of presentation to the hummingbird: once the bird had fed from the feeder, the first whiteboard was presented. On this board the orange flower at the bottom contained reward, while on the board that followed the bird would find reward only in the purple flower at the top of the board. The feeder would then be returned and the next trial would begin. From Jelbert *et al*. [28].

Jelbert *et al*.’s [28] experiment was based on a scene-learning paradigm used to test the ability for rhesus macaques *Macaca mulatta* to learn the visual and spatial features of both rewarded and unrewarded items in a scene [44]. In both experimental conditions, free-living hummingbirds were presented with two consecutive boards, each bearing four flowers (figure 2). Two of the flowers were one colour (e.g. pink), and the other two flowers were another colour (e.g. green). The pairs of differently coloured flowers were placed either at the top or the bottom of the boards. Only one flower on each board was rewarded. In the first condition (the so-called ‘visual’ condition) each board was of a different colour pattern, and the colour pattern of the board was the cue as to which flower contained the reward, both its location and its colour. For example, on the board with the blue stripes, the pink flower at the top of the board contained the reward. This left the pink flower at the bottom of the board and both green flowers (top and bottom) without reward. When the boards were presented the next time, the reward was to be found in the green flower at the bottom of the yellow board. In the second condition (the ‘sequential’ condition), the boards were not differentiated by colour pattern (both white) and the birds had to learn which of the four flowers on a board was rewarded using the alternating sequence of board presentation. For example, the rewarded flower on the first board they encountered was the orange one at the bottom, and on the following board it was the green flower at the top. As in the experiment described above [27], this experiment did not look for one-time memories but for memories in which three different components needed to be integrated for the bird to do best. Again, the design enabled examination of the errors the birds made. In this different version of a what—where—when task, the birds were most likely to make the correct choice than to make any other decision. Their performance did not differ between the visual and the sequential condition, although they were faster to learn the visual than the sequential condition. Their errors did differ across the conditions, however, as they made more ‘which’ errors in the sequential condition than any other error type in either condition. That they learned the rules of the task as the experiment proceeded was shown by their distribution of errors in the first 10 trials versus those in the last 10 trials: in the first 10 trials, birds made errors that were equally distributed across the three types of error (what, where and which) but by the last 10 trials they were making a small handful of what errors and all their other errors were which errors.

While one might question the ecological value of the ‘which’ component of this task to a wild hummingbird [45], hummingbirds do experience flowers in different contexts. For example, different patches of flowers in different parts of the territory, in different places along a migration route or at different times of the breeding season.

What both the Marshall *et al*. [27] and Jelbert *et al*. [28] experiments showed was the capacity of animals in the wild to integrate the three components of an episodic memory and to do so when they are also busy about the rest of their daily lives: these birds were displaying to females and to rivals, defending their territories and more while participating in these experiments. However, these memories were not for single events, and indeed, it would seem far from useful for one of these hummingbirds to retain any one of these beyond the context of the experiment, which in the Marshall *et al.* experiment was for a few days, and in the Jelbert *et al.* experiment for even less time.

## 4. The temporal component of memory

Both hummingbird experiments also showed the relatively greater difficulty these animals had with remembering the temporal or context component that would lead them to the reward than remembering either its colour or its location. Temporal information presents a variety of challenges particularly for the hunt for episodic memory, not least because it comes in a number of forms. The time an event occurred could be encoded as part of an annual cycle, which for animals may be seasonal, part of a daily cycle, such as time of day, which for wild animals is associated with the location and movement of the sun across the sky, or as time that has elapsed since the event. Sequences also allow for encoding the temporal component of a memory, such as remembering an event as is the one that preceded or followed another event. It is perhaps useful, however, before we go looking for temporal capabilities, to consider what abilities natural selection might have favoured, as well as the possible outcomes of selection. For example, it is not clear that it would be beneficial for an animal to remember when it was born or even which year, in which an event occurred. However, being able to learn when in the year or when in a day, similar events are likely to occur in the future does seem useful, as does remembering how long ago an event occurred (e.g. hummingbirds foraging on flowers that refill) and the sequence of events, such as whether a particular interaction with conspecific occurred before or after an interaction with another individual [46].

There are plenty of data to show the value and role of annual/seasonal timing of events for wild animals; it is clear that natural selection has led to a variety of responses to cues that signal such passing of time, which do not involve memory. For example, in many vertebrates, an individual’s hormonal levels respond to changes in ambient light levels and bring an animal that breeds seasonally into reproductive state or to fatten in preparation for migration at an appropriate time: ‘planning’ as enabled by natural selection.

There is also a wealth of data to show that animals learn to associate the time of day with events, good or bad. This is also a mechanism shaped by natural selection and found almost universally. It has been put to useful experimental effect in a plethora of clock-shifting experiments, as has often been used in the investigation of navigation or spatial memory abilities (e.g. [47–49]).

The first Marshall *et al*. [27] experiment showed that hummingbirds could learn when a particular flower in a particular location would contain reward. However, it was not clear whether they had learned ‘when’ as the time of day the array was presented or as a part of a sequence, i.e. when the array is first presented in a day (in the morning), choose one flower, but when it is presented for the second time in the day (in the afternoon), choose the other flower associated with reward. The follow-up experiment showed that animals might learn and then combine more than one type of temporal information: in the case of the hummingbirds, in a test condition where sequence and time of day were put in conflict, the birds appeared to use time of day to determine which sequence rule to apply. In that case, if the array was presented earlier in the day than was usual, the birds appeared to know that it was being presented earlier and so chose the flower that was rewarded at the other time of day; they also made this response when the array was presented to them later than was usual. To make the appropriate response in both test conditions required them to use both the time of day and the sequence in which arrays had been presented. How often do we find that animals are capable of doing something more than we expected of them?

That wild animals learn sequences has been described since at least Darwin, who observed bees traplining around flowers as he took his daily walk. Traplining is a foraging behaviour in which animals regularly repeat the sequence in which they move around a number of resources. To be efficient, traplining does not just require memory for the locations of resources but also when they were last visited to ensure the highest probability that the resource at each location has been renewed. Pollinators have proved to be the most typical group of animals to examine traplining, and there are plenty of data to show not just hummingbirds but bumble and honey bees [50–52] are efficient trapline foragers. Both taxa update their traplines as resources change, no longer visiting locations when they become less rewarding [53]. Hummingbirds, at least, respond asymmetrically to changes in the resources in their trapline: they respond sooner and more strongly to decreases in reward quality than to increases. The key point here, however, is that these animals regularly have to remember resources in a sequential fashion, but also not to remain bound to any sequence, should the resources change [54,55].

## 5. The value of integration of information

While none of the preceding examples of memory abilities of animals in the wild provide evidence specifically for episodic memory, they do repeatedly demonstrate that integration of information into single memories is commonplace: animals are not just learning the location of a reward, or what it looks like. The early fieldwork on cognition in bees, hummingbirds and a variety of other animals consisted of determining memory capabilities that focussed on a singular ability, such as spatial memory capability or visual cue use (e.g. [33,56–58]). More recently, however, the use of memories that involve integration of information by wild animals is increasingly evident e.g. chacma baboons resume foraging routes after interruptions [59], time-place learning by rufous hummingbirds *Selasphorus rufus* [60] and ants [61]. Gradually, we are gaining access to increasingly complex cognitive abilities of animals, even in the wild. The demonstration of episodic memory in food-storing scrub jays was, arguably, the key stimulus to these discoveries. However, there is no reason to believe that episodic memory is restricted to just a few special taxa as the advantage of remembering an integration of what happened, where it happened and when it happened can be argued for a variety of ecological scenarios. It seems at this point, however, that it is our ability to test for this kind of memory in wild animals that is restricted. Here, we have proposed a way to approach this restriction: if we assume that episodic memories lie at the base of all memories, perhaps we can track back, either theoretically or empirically, to an animal’s initial experience. Then, we might ask if that experience was an integration of a what, a where and a when, from which less relevant information was dropped. Collecting these data from the wild would not be technically trivial but would be at least plausible.

## Data Availability

This article has no additional data.
